# Evaluation of Antioxidant Function of Palygorskite and Its Derivatives In Vitro and for Broilers

**DOI:** 10.3390/antiox14101181

**Published:** 2025-09-27

**Authors:** Tie Gao, Shengjia Wang, Yiding Yang, Jibo Li, Yanmin Zhou

**Affiliations:** College of Animal Science and Technology, Nanjing Agricultural University, Nanjing 210095, China; 2022205044@stu.njau.edu.cn (T.G.);

**Keywords:** palygorskite, antioxidant capacity, broilers

## Abstract

This study aimed to investigate the antioxidant properties of natural palygorskite (N-pal), bundle-dissociated palygorskite (D-pal), and zinc-bearing palygorskite (Zn-pal), as well as their effects on the antioxidant capacity of broilers. Palygorskite (Pal) and its derivatives exhibited hydrogen peroxide (H_2_O_2_) decomposition and hydroxyl radical (·OH) scavenging abilities, with D-pal and Zn-pal demonstrating superior performance. A total of 320 one-day-old Arbor Acres broilers were randomly divided into 4 groups: control, N-pal, D-pal and Zn-pal groups. The corn–soybean meal basal diet was supplemented with N-pal (10 g/kg), D-pal (5 g/kg) and Zn-pal (1 g/kg) for the respective treatment groups. The trial lasted for 42 days. At 21 days, D-pal and Zn-pal groups significantly increased average body weight and average daily weight gain while reducing the feed-to-gain ratio (*p* < 0.05). Both reactive oxygen species (ROS) and malondialdehyde (MDA) levels in liver of broilers were significantly reduced, and glutathione peroxidase 1 (GPX1) gene expression was upregulated at 21 days (*p* < 0.05). N-pal and D-pal groups enhanced superoxide dismutase (SOD) and catalase (CAT) activities in the duodenum (21d); D-pal group increased SOD activity in the ileum (42d); all Pal groups decreased ileal jejunal mucosal ROS (21d) and MDA (42d) (*p* < 0.05). At 42 days, Pal supplementation downregulated oxidative stress (N-pal), oxidoreductase activity (D-pal), and H_2_O_2_ response (Zn-pal) pathways in jejunal mucosa. N-pal and D-pal groups upregulated ileal mucosal nuclear factor-erythroid 2-related factor-2 (Nrf2), heme oxygenase-1 (HO-1), and superoxide dismutase 1 expression, while Zn-pal group increased HO-1 expression (21d). D-pal and Zn-pal groups enhanced jejunal mucosal Nrf2 and HO-1 expression (21d). Pal improved the antioxidant capacity in broilers by activating the Nrf2-mediated pathway, upregulating antioxidant-related gene expression, particularly at 21 days. Both D-pal and Zn-pal demonstrated potent antioxidant efficacy, and they can improve growth performance by enhancing the animal’s antioxidant capacity.

## 1. Introduction

Broilers provide high-quality animal protein for the global population. However, poultry production is frequently affected by various environmental stressors, such as high temperatures, high stocking density, transportation, and nutritional deficiencies [1]. Against the backdrop of global climate change [2,3], poultry is becoming increasingly vulnerable to oxidative stress caused by high ambient temperatures [4,5,6]. These multifaceted stressors underscore the urgent need to enhance antioxidant capacity in modern poultry production systems. Oxidative stress occurs when the activity of oxidants exceeds the intrinsic antioxidant defenses of an organism, resulting in varying degrees of oxidative damage. This process can induce cellular DNA and protein damage, lipid peroxidation, and apoptosis [3,7,8]. This phenomenon arises from excessive peroxides and free radicals within the organism—particularly reactive oxygen species (ROS) [9]. Oxidative stress occurs when these radicals overwhelm the scavenging capacity of poultry’s antioxidant enzymes [10]. Under oxidative stress conditions, excessive free radicals attack biological membranes in broilers, leading to an imbalance in intracellular redox homeostasis. This disruption impairs digestion and nutrient absorption, increases the risk of intestinal disorders, and ultimately leads to a decline in growth performance [11]. Rising living standards have led to increasingly stringent consumer demands for meat quality. However, oxidative stress suppresses cellular antioxidant capacity in broilers, and accumulated ROS oxidize unsaturated fatty acids and structural proteins, ultimately leading to deterioration in meat quality [12]. To mitigate the impact of oxidative stress, dietary antioxidants and micronutrients are commonly supplemented in broiler feed.

Clay minerals exhibit significant intrinsic antioxidant capacity, with kaolin demonstrating efficient scavenging activity against extracellular reactive oxygen species (ROS) [13]. Palygorskite (Pal) is a magnesium aluminum silicate mineral with a 2:1 layered chain structure [14]. It serves as a natural one-dimensional nanomaterial. Natural palygorskite (N-pal) exhibits a microstructure comprising rod-shaped crystals (rod-crystals), bundles of rod-crystals, and aggregated clusters. Its unique pore structure and cation exchange capacity confer exceptional adsorptive, colloidal, and carrier properties, enabling widespread applications in materials science, environmental engineering, and biological/medical fields [15,16,17]. Many Pal composite materials and their products also have good antioxidant properties. Cu/Palygorskite composite exhibits strong antioxidant activity [18]. Moreover, the complexation of Pal with curcumin enhances the antioxidant activity of curcumin alone [19]. Furthermore, Co_3_O_4_ nanoparticles were used to modify Pal via hydrothermal synthesis, and the resulting nanocomposite functions as a nanozyme exhibiting enhanced peroxidase-like activity, enabling the construction of a highly sensitive and selective colorimetric sensor for H_2_O_2_ detection [20]. Pal can improve the antioxidant function of poultry. Dietary supplementation with Pal alleviates in vivo oxidative damage and mitigates oxidative stress in broilers [21]. In laying hens, dietary supplementation with N-pal improves intestinal morphology and digestive function [22]. Beyond N-pal, composite materials utilizing Pal as a functional carrier demonstrate significant potential for antioxidant applications in poultry production. The zinc-oxide-loaded Pal composite material improved the antioxidant status of broilers [23]. The essential oil/palygorskite composite improved the antioxidant capacity of laying hens [24]. Although these results indicate that Pal possesses intrinsic antioxidant potential and enhances animals’ antioxidant capacity, its antioxidative effects remain insufficiently studied. Therefore, we investigated the antioxidant properties of Pal and its derivatives by assessing the antioxidant capacity of broilers and employing transcriptomic analysis to elucidate the expression patterns of antioxidant-related genes.

## 2. Materials and Methods

The N-pal and bundle-dissociation palygorskite (D-pal) samples were provided by Jiangsu Jinhan New Materials Co., Ltd. (Xuyi, China). The D-pal sample was prepared from N-pal using ultrasonic-assisted crystal bundle dissociation technology [25]. The zinc-bearing palygorskite (Zn-pal) was synthesized according to our laboratory’s established protocol: N-pal was activated by calcination in a chamber electric furnace (SX2-4-10N, China) at 300 °C for 3 h. The activated material was then uniformly mixed with ZnCl_2_ at a 4:1 weight ratio (*w*/*w*) and recalcined at 300 °C for 3 h. After cooling, the product was washed with deionized water to remove unbound Cl^−^ and Zn^2+^ ions, oven-dried at 105 °C for 5 h (Electric thermostatic drying oven, GZX-9240MBE, Shanghai Boxun Medical Bio-Company Ltd., Shanghai, China), and finally ground to pass through a 200-mesh sieve [26]. The zinc content was determined to be 21.16 mg/g using inductively coupled plasma optical emission spectrometry (ICP-OES, ICAP 7000, Thermo Fisher Scientific, Waltham, MA, USA) with matrix-matched calibration standards to ensure measurement accuracy. The major chemical composition of Pal was determined by X-ray fluorescence (XRF) spectrometry using an Epsilon 4 spectrometer (PANalytical B.V. Almelo, Netherlands). Powdered samples were pressed into pellets and analyzed under helium atmosphere for N-pal, including SiO_2_ (56.69%), Al_2_O_3_ (6.89%), Fe_2_O_3_ (5.21%), CaO (1.72%), MgO (8.80%), K_2_O (0.96%) and Na_2_O (1.19%); for D-pal, including SiO_2_ (40.47%), Al_2_O_3_ (8.10%), Fe_2_O_3_ (7.04%), CaO (9.49%), MgO (8.04%), K_2_O (1.78%) and Na_2_O (1.21%). According to published data from our laboratory, the optimal dietary supplementation levels of N-pal, D-pal, and Zn-pal for broilers aged 1 to 42 days were determined as 10 g/kg, 5 g/kg, and 1 g/kg, respectively [25,27,28].

### 2.1. Hydrogen Peroxide and Hydroxyl Radical Scavenging Capacity of Pal and Its Derivatives

The ability of Pal to scavenge hydrogen peroxide (H_2_O_2_) was assessed by measuring the decrease in absorbance of H_2_O_2_ at 240 nm using a UV spectrophotometer (MAPADA, UV-1200, Shanghai, China). The absorbance at 240 nm was measured over 25 min for the following samples: H_2_O_2_ (10 mM) alone (control), N-pal (0.25, 0.50, 0.75 and 1.00 mg/mL) + H_2_O_2_ (10 mM), D-pal (0.25, 0.50, 0.75 and 1.00 mg/mL) + H_2_O_2_ (10 mM) and Zn-pal (0.25, 0.50, 0.75 and 1.00 mg/mL) + H_2_O_2_ (10 mM). Double-distilled water was used as blank for zero adjustment. The H_2_O_2_ scavenging rate was calculated using the following formula:

H_2_O_2_ scavenging rate (%) = [(A_control_ − A_sample_)/A_control_] × 100

The hydroxyl radical (·OH) scavenging activity of Pal was determined using a commercial assay kit (Cat. No.: A018-1-1, Nanjing Jiancheng Bioengineering Institute, Nanjing, China) according to the manufacturer’s instructions.

### 2.2. Animals, Diets, and Management

The animal study protocol was approved by the Institutional Animal Care and Use Committee of Nanjing Agricultural University (Certification No.: NJAU.No20240914N22).

This experiment employed a completely randomized single-factor design. A total of 320 one-day-old Arbor Acres broilers with an average body weight of 41.6 ± 0.16 g were randomly allocated into 4 groups. Each group contained 8 replicates (cages) with 10 chicks. The control group was fed the basal diet, while the treatment groups received supplemental N-pal (10 g/kg of feed), D-pal (5 g/kg of feed) and Zn-pal (1 g/kg of feed). A corn–soybean meal basal diet was prepared according to the NRC (1994) [29]. The compositions of the basal diet and its nutrient levels are presented in Table 1. The trial was conducted over a 42-day period, covering the broiler starter (days 1–21) and grower (days 22–42) phases. During the trial, all broilers were provided with feed and water ad libitum under a lighting schedule of 23 h of light and 1 h of darkness, except for the first three days, when maintained under constant lighting conditions (24 h/d). All broilers were vaccinated according to standard commercial protocols. The ambient temperature was maintained at 33–34 °C for the first 3 days and subsequently reduced by 2 °C weekly until reaching 22 °C. Relative humidity was set at 60 to 70% during the first week and was maintained at 50 to 60% thereafter.

### 2.3. Sample Collection

At 21 and 42 days of age, two birds per replicate pen with body weights closest to the group mean were selected. Whole blood samples were collected from the wing vein using anticoagulant tubes. Serum was obtained by centrifugation (Eppendorf, Centrifuge 5804 R, Eppendorf AG, Hamburg, Germany) at 4 °C (3500 r/min, 10 min) and stored at −20 °C pending analysis. Birds were euthanized by cervical dislocation. The abdominal cavity was immediately opened to excise the liver. Segments of the small intestine (duodenum, jejunum and ileum) were isolated, longitudinally incised, and mucosal samples scraped using sterile glass microscope slides. All tissues were flash-frozen in liquid nitrogen and stored at −80 °C (Thermo Fisher, TDE50086FV-ULTS, USA) until further processing.

### 2.4. Growth Performance

Following grouping at 1 day of age, broilers were weighed by replicate. Subsequent weights were recorded after 12 h fasting at 21 and 42 days, with feed intake monitored throughout the trial to calculate growth performance parameters, including average body weight (ABW), average daily weight gain (ADG), average daily feed intake (ADFI), and feed-to-gain ratio (F/G).

### 2.5. Detection of Reactive Oxygen Species in the Liver and Jejunal Mucosa of Broilers

Liver and ileal mucosa tissues from 21- and 42-day-old broilers were subjected to ROS detection using the fluorescent probe dihydroethidium (DHE) via a commercial ROS assay kit. The ROS Assay Kit (Beyotime Biotechnology, Cat. No.: S0064S, Shanghai, China) was employed according to the manufacturer’s instructions. Fluorescence was detected using a fluorescence microscope (Nikon, ECLIPSE 80i, Tokyo, Japan) at excitation/emission wavelengths of 535 nm and 610 nm, respectively, to determine intracellular ROS levels.

### 2.6. Antioxidant Capacity of Broilers

The levels of antioxidant-related parameters in the serum, liver, and intestinal mucosa of broilers at 21 and 42 days of age were measured using commercially available assay kits. Malondialdehyde (MDA, Cat. No.: A003-1-2), superoxide dismutase (SOD, Cat. No.: A001-1-2), glutathione peroxidase (GSH-Px, Cat. No.: A005-1-2), catalase (CAT, Cat. No.: A015-1-1), and total antioxidant capacity (T-AOC, Cat. No.: A015-1-1) were measured using assay kits provided by the Nanjing Jiancheng Bioengineering Institute (Nanjing, China), in accordance with the manufacturer’s instructions.

### 2.7. Quantitative Real-Time PCR (qRT-PCR)

To validate the RNA-seq results, five antioxidant-related genes were selected for expression analysis in liver, jejunal, and ileal mucosal tissues of broilers at 21 and 42 days of age. The total RNA was extracted using a commercial RNA extraction kit (Accurate Biology, Catalog No. AG21024, Changsha, China). Subsequently, RNA was converted to complementary DNA (cDNA) via reverse transcription with the PrimeScript™ RT reagent Kit (TaKaRa, Dalian, China). cDNA was analyzed by real-time PCR using 5X All-In-One RT MasterMix (abmGood, Cat. No.: G592, Zhenjiang, China). The primer information is shown in Table 2. The 2^−ΔΔCt^ method was used for the statistical analysis of qPCR data [30].

### 2.8. RNA Extraction, Library Construction and Sequencing

Total RNA was isolated from jejunal mucosal tissues using TRIzol reagent (Accurate Biology, Cat. No.:BS258A, Changsha, China) according to the manufacturer’s protocol. The quality and quantity of the extracted RNA were assessed using a NanoDrop ND-1000 UV spectrophotometer (NanoDrop Technologies, Wilmington, DE, USA) to determine the 260 nm/280 nm absorbance ratio and using an Agilent 2100 bioanalyzer (Agilent Biotechnologies, Palo Alto, CA, USA) to evaluate RNA integrity. The RNA integrity was subsequently verified by electrophoresis on RNase-free agarose gels. Construction of the cDNA library and RNA-seq were performed by Bena Technology Co. Ltd. (Bena Technology, Wuhan, China). RNA-seq libraries were sequenced on an Illumina NovaSeq 6000 platform (Illumina, San Diego, CA, USA) to generate 150 bp paired-end reads. Raw sequencing data were quality-filtered using fastp [31] to obtain clean reads, followed by quality verification with FastQC (Version: 0.11.9; Parameters: default) [32]. Clean reads were aligned to the reference genome using STAR (Version: 2.7.9a; Parameters: default) [33], followed by alignment statistics. The positional distribution of sequencing reads across genes was analyzed to evaluate mRNA fragmentation randomness. Gene/transcript expression levels were quantified using RSEM (RNA-Seq by Expectation-Maximization) [34] by calculating FPKM (Fragments Per Kilobase per Million bases). DESeq2 (Version: 1.48.2) [35] was utilized to identify differentially expressed genes (DEGs) between groups, using thresholds of |Log2(FC)| ≥ 1 and *p* < 0.05. Gene Set Enrichment analysis (GSEA), Gene Ontology (GO) and Kyoto Encyclopedia of Genes and Genomes (KEGG) functional enrichment analysis were performed using clusterProfiler (Version: 3.14.3) [36].

## 3. Statistical Analysis

Statistical analyses were conducted using SPSS 22.0 software; all the data were analyzed by a one-way ANOVA, and differences among groups were tested using Duncan’s multiple range tests. *p* < 0.05 was considered statistically significant. All experimental data were expressed using the mean ± standard error of the mean (SEM).

## 4. Results

### 4.1. Hydrogen Peroxide and Hydroxyl Radical Scavenging Capacity of Pal and Its Derivatives

**Figure 1:**
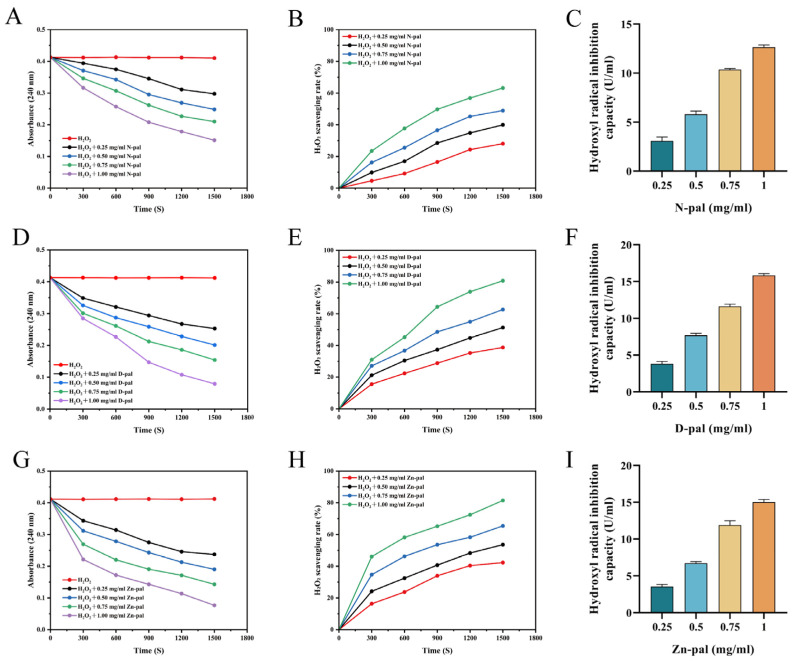
Hydrogen peroxide and hydroxyl radical scavenging capacity of Pal and its derivatives. Plot of absorbance versus time of H_2_O_2_ (**A**,**D**,**G**). H_2_O_2_ scavenging rate (**B**,**E**,**H**). Hydroxyl radical inhibition (**C**,**F**,**I**). Results are the average values of triplicate measurements.

As shown in Figure 1A,D,G, Pal exhibited concentration-dependent H_2_O_2_ scavenging activity. At 1.00 mg/mL, N-pal could scavenge over 60% of H_2_O_2_, while both D-pal and Zn-pal at the same concentration removed more than 80% of H_2_O_2_. Pal also demonstrated concentration-dependent ·OH scavenging activity. The results ruled out the possibility that H_2_O_2_ might generate hydroxyl radicals under Pal treatment, indicating that Pal’s H_2_O_2_ scavenging capacity can be attributed to its intrinsic antioxidant properties.

### 4.2. Growth Performance

Compared with the CON group, the ABW and ADG were significantly increased in the D-pal and Zn-pal groups during days 1–21 (*p* < 0.05), while the N-pal, D-pal, and Zn-pal groups significantly reduced the F/G (*p* < 0.05) (Table 3).

### 4.3. Detection of Reactive Oxygen Species in the Liver and Jejunal Mucosa of Broilers

**Figure 2:**
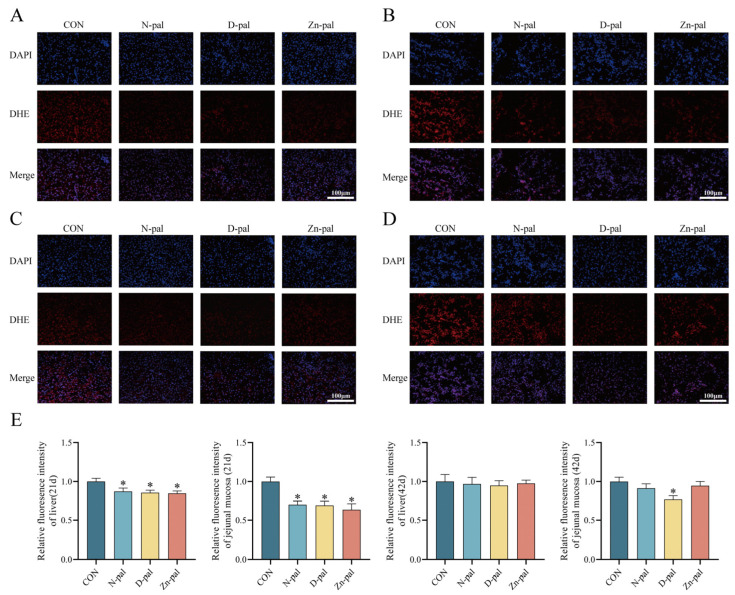
Effects of Pal and its derivatives on ROS staining and relative fluorescence intensity in liver and jejunum tissues of broilers. At 21 days: liver (**A**), jejunal mucosa (**B**). At 42 days: liver (**C**), jejunal mucosa (**D**). Relative fluorescence intensity (**E**). * Means significant difference from the CON group (*p* < 0.05). Nuclear staining with DAPI (blue). Superoxide detection using DHE (red); intensity reflects ROS levels. Merged image showing co-localization of DAPI and DHE signals.

Compared with the control group, ROS levels in all treatment groups were significantly reduced in both liver tissue and jejunal mucosa at 21 days of age (*p* < 0.05). At 42 days of age, the D-pal group exhibited significantly decreased ROS levels in the jejunal mucosa (*p* < 0.05), while no significant differences were observed in the other groups, as shown in Figure 2.

### 4.4. Antioxidant Capacity of Broilers

At 21 days of age, the serum supplemented with Pal in the diet significantly increased the activities of GSH-Px and SOD, and significantly reduced the MDA level (*p* < 0.05). The level of MDA in liver tissue was significantly reduced in each treatment group (*p* < 0.05). In the duodenum, the activities of SOD and CAT were significantly increased in the N-pal, D-pal and Zn-pal groups (*p* < 0.05). The jejunal treatment significantly increased CAT activity (*p* < 0.05); In the ileum, N-pal and D-pal significantly increased the activities of SOD and CAT, while the N-pal and Zn-pal groups significantly decreased the level of MDA (*p* < 0.05) (Table 4 and Table 5). These results indicate that dietary supplementation with N-pal, D-pal, and Zn-pal enhances antioxidant enzyme activity in broilers at 21 days of age.

At 42 days of broilers, the levels of MDA in serum and ileum were significantly reduced in each treatment group, and the activity of SOD in the ileum was significantly increased in the D-pal group (*p* < 0.05) (Table 6 and Table 7).

### 4.5. Expression of Antioxidant-Related Genes in Liver and Jejunal Mucosa

**Figure 3:**
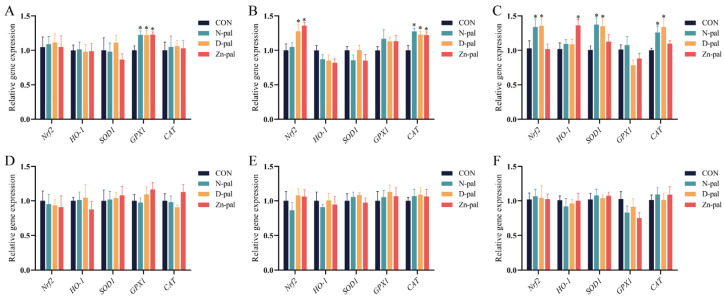
Effects of dietary Pal and its derivatives supplementation on antioxidant-related gene expression in liver and jejunal mucosa of broilers. Nrf2, nuclear factor-erythroid 2-related factor-2; HO-1, heme oxygenase-1; SOD1, superoxide dismutase 1; GPX1, glutathione peroxidase 1; CAT, catalase. At 21 days: liver (**A**), jejunal mucosa (**B**), ileal mucosa (**C**). At 42 days: liver (**D**), jejunal mucosa (**E**), ileal mucosa (**F**). * Means significant difference from the CON group (*p* < 0.05).

At 21 days of age, dietary supplementation with Pal significantly increased the expression of the GPX1 gene in the liver compared to the control group (*p* < 0.05); the expression levels of Nrf2 and HO-1 in the D-pal and Zn-pal groups were significantly increased in the jejunal mucosa (*p* < 0.05), with each treatment group showing increased CAT gene expression (*p* < 0.05); in the ileal mucosa, the expression levels of Nrf2, HO-1, and SOD1 in the N-pal and D-pal groups were significantly increased (*p* < 0.05), and the expression of the HO-1 gene in the Zn-pal group was significantly increased (*p* < 0.05). At 42 days of age, the addition of Pal had no significant effect on the antioxidant-related genes in the liver and jejunal mucosa of broiler chickens (*p* > 0.05), as shown in Figure 3.

### 4.6. Transcriptome Analysis Results

**Figure 4:**
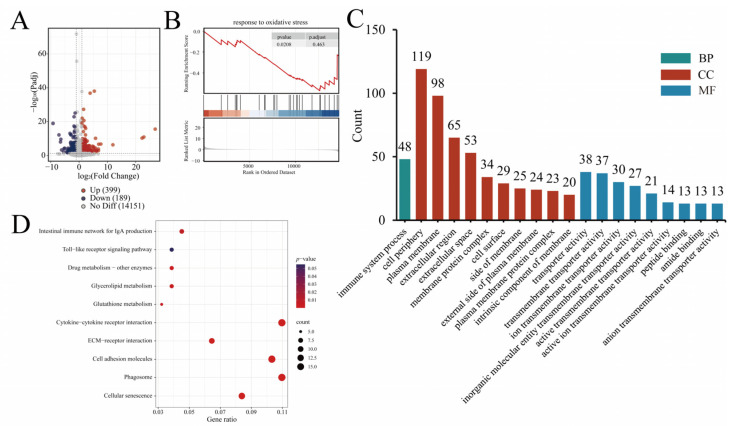
RNA-seq analysis (N-pal vs. CON). (**A**) Volcano plot; (**B**) GESA GO plot; (**C**) GO enrichment analysis results; (**D**) KEGG enrichment pathway. In the GSEA GO plot, red and blue represent genes with high expression in the experimental and control groups, respectively. Bar plot of GO enrichment analysis with the number of enriched genes indicated.

**Figure 5:**
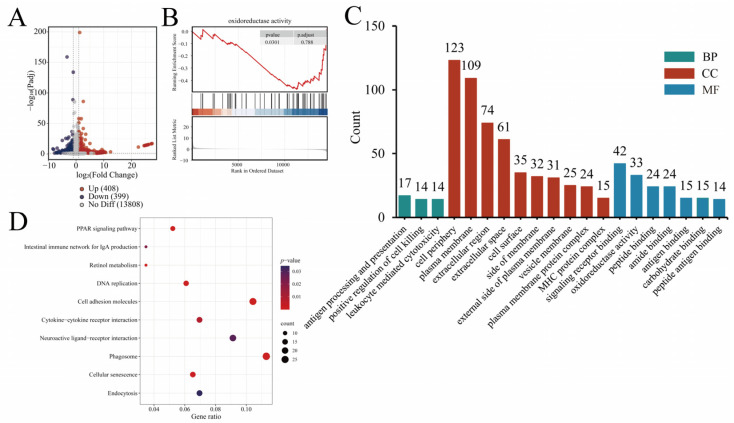
RNA-seq analysis (D-pal vs. CON). (**A**) Volcano plot; (**B**) GESA GO plot; (**C**) GO enrichment analysis results; (**D**) KEGG enrichment pathway. In the GSEA GO plot, red and blue represent genes with high expression in the experimental and control groups, respectively. Bar plot of GO enrichment analysis with the number of enriched genes indicated.

**Figure 6:**
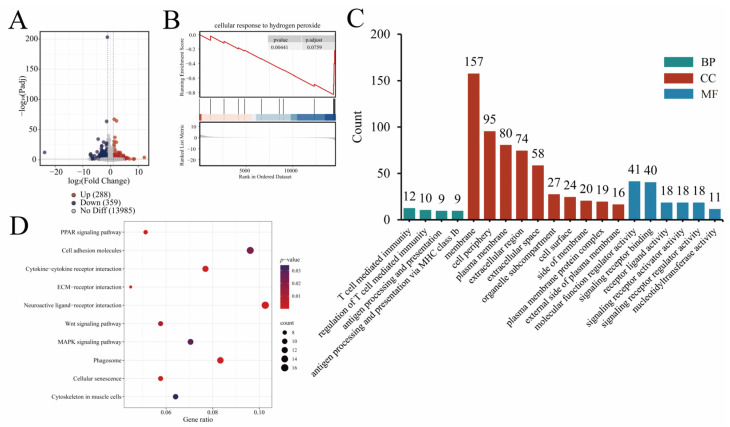
RNA-seq analysis (Zn-pal vs. CON). (**A**) Volcano plot; (**B**) GESA GO plot; (**C**) GO enrichment analysis results; (**D**) KEGG enrichment pathway. In the GSEA GO plot, red and blue represent genes with high expression in the experimental and control groups, respectively. Bar plot of GO enrichment analysis with the number of enriched genes indicated.

Transcriptome sequencing was performed on 24 cDNA libraries to investigate differences in jejunal mucosal tissue between treated and control groups of 42-day-old broilers, with 6 RNA-Seq samples per group. The transcriptome results of this study showed that compared with the control group, the N-pal, D-pal and Zn-pal groups, respectively, detected 588, 807 and 647 differentially expressed genes. The transcriptome results of this study showed that, compared with the control group, the N-pal, D-pal, and Zn-pal groups had 588, 807, and 647 differentially expressed genes, respectively. Among these, 399, 408, and 288 genes were upregulated (Figure 4A, Figure 5A and Figure 6A). KEGG analysis showed that the differentially expressed genes in the N-pal group were significantly enriched in intestinal immune network for IgA production, Toll-like receptor signaling pathway, Drug metabolism—other enzymes, Glycerolipid metabolism, Glutathione metabolism, Cytokine–cytokine receptor interaction, ECM–receptor interaction, Cell adhesion molecules, Phagosome and Cellular senescence pathways. Differentially expressed genes in the D-pal group were significantly enriched in the PPAR signalling pathway, intestinal immune network for IgA production, retinol metabolism, DNA replication, cell adhesion molecules, cytokine–cytokine receptor interaction, neuroactive ligand–receptor interaction, phagosome, cellular senescence, and endocytosis pathways. Differentially expressed genes in the Zn-pal group were significantly enriched in the PPAR signaling pathway, Cell adhesion molecules, Cytokine–cytokine receptor interaction, ECM–receptor interaction, Neuroactive ligand–receptor interaction, Wnt signaling pathway, MAPK signaling pathway, Phagosome, Cellular senescence and Cytoskeleton in muscle cell pathways (Figure 4D, Figure 5D and Figure 6D).

According to the biological process (BP) analysis, all treatment groups were primarily associated with cellular immune responses. Specifically, the N-pal group was enriched in immune system processes, the D-pal group showed significant enrichment in positive regulation of cell killing and antigen processing and presentation, while the Zn-pal group was predominantly associated with positive regulation of T cell-mediated immunity. Cellular component (CC) analysis demonstrated significant enrichment in membrane-associated and extracellular compartments, including the cell periphery, plasma membrane, extracellular space and extracellular region, in each treatment group. Molecular function (MF) analysis indicates that in the N-pal group, transporter activity is dominant, including transmembrane transporter activity, ion transmembrane transporter activity, and inorganic molecular entity transmembrane transporter activity; in the D-pal group, the dominant molecular functions are signaling receptor binding and oxidoreductase activity, including peptide binding, antigen binding and carbohydrate binding; in the Zn-pal group, the dominant molecular functions are molecular function regulator activity and signaling receptor binding, including receptor ligand activity, signaling receptor activator activity and signaling receptor regulator activity (Figure 4C, Figure 5C and Figure 6C).

GSEA revealed significant downregulation of key pathways in jejunal mucosa at 42 days of age (Figure 4B, Figure 5B and Figure 6B), including response to oxidative stress (N-pal), oxidoreductase activity (D-pal), and cellular response to hydrogen peroxide (Zn-pal).

## 5. Discussion

H_2_O_2_, as a ROS in biological systems, is a mediator of oxidative stress. Excessive H_2_O_2_ can lead to oxidative damage, causing lipid peroxidation, DNA mutations, and protein denaturation, which are associated with aging, cancer, and other diseases [37,38,39,40]. The ability to decompose hydrogen peroxide is a key indicator for evaluating antioxidants. The present study demonstrates that Pal exhibits significant concentration-dependent scavenging activity against both H_2_O_2_ and hydroxyl radicals. Our findings reveal distinct efficiency patterns among the different Pal variants, with N-pal showing over 60% H_2_O_2_ removal and both D-pal and Zn-pal exceeding 80% removal at the highest tested concentration (1.00 mg/mL). Studies have shown that Pal can inhibit lipid peroxidation, and its dose–response relationship suggests that this inhibition is surface-mediated [41]. The differences in scavenging efficiency observed in this study may be attributed to varying surface active site exposure resulting from different modification methods (e.g., D-pal and Zn-pal). The observed concentration-dependent activity suggests that Pal’s antioxidant capacity follows a dose–response relationship, a characteristic feature of many natural antioxidants [42,43]. The superior performance of D-pal and Zn-pal compared to N-pal indicates that specific modifications can enhance Pal’s redox-active properties, possibly through the introduction of additional electron-donating groups or improved surface characteristics that facilitate electron transfer reactions [44,45]. The ·OH is a highly reactive free radical with strong oxidative capacity. The Fenton reaction can decompose H_2_O_2_ to generate ·OH [46]. Importantly, our results exclude the possibility that H_2_O_2_ decomposition leads to hydroxyl radical generation in the presence of Pal. This finding is significant because it demonstrates that Pal functions as a direct antioxidant rather than a pro-oxidant that might inadvertently increase oxidative stress through radical chain reactions [47,48]. The ability to scavenge both H_2_O_2_ and hydroxyl radicals suggests that Pal possesses multiple antioxidant mechanisms, potentially including direct reduction, radical stabilization, and possibly metal chelation.

Multiple studies have documented the growth-promoting effects of Pal in poultry [23,49]. In the present study, during the starter phase (days 1 to 21), the D-pal and Zn-pal groups exhibited significant increases in ABW and ADG, while all Pal-supplemented groups showed a reduced F/G ratio. These findings indicate that structural modifications—specifically bundle dissociation and zinc loading—effectively enhance the biofunctionality of Pal beyond its native state. Based on an integrated understanding of the biological functions of Pal and zinc in animals, the observed effects can be mechanistically explained. The significant reduction in F/G and improved nutrient utilization efficiency across all treatment groups aligns with Pal’s established capacity to adsorb molds and mycotoxins, as well as to enhance intestinal barrier function [50,51]. The superior growth performance of D-pal compared to N-pal indicates that bundle dissociation increases the specific surface area, thereby enhancing the capacity to adsorb mycotoxins from feed. The zinc-mediated metabolic enhancement suggests that the significant improvement in starter-phase ADG with Zn-pal is associated with zinc’s critical roles in protein synthesis and carbohydrate metabolism [52,53,54]. This study revealed that the N-pal group showed no significant improvement in ABW or ADG during days 1–21, despite a reduced F/G, indicating that N-pal primarily enhances feed efficiency rather than growth kinetics. This contrasts with the modified variants, D-pal and Zn-pal, which actively promote anabolic processes. Notably, this study demonstrates that Pal supplementation significantly enhances antioxidant function in broilers, with particularly pronounced effects during the 1–21-day phase. The response hierarchy, Zn-pal ≥ D-pal > N-pal, reflects the following: N-pal’s efficacy as an adsorbent improves nutrient utilization; D-pal’s optimized morphology enhances nutrient retention; and Zn-pal exhibits dual functionality by combining toxin adsorption with essential micronutrient provision. These findings support the hypothesis that physical and chemical modifications potentiate the bioactive growth-promoting properties of Pal beyond its native state.

The multitissue improvements in antioxidant biomarkers—particularly the temporospatial specificity of structural modifications—reveal complex redox modulation mechanisms. Clay minerals such as phyllosilicates and zeolites have been demonstrated as effective in vitro radical scavengers [55,56], implicating that Pal may exhibit potent radical scavenging capacity due to similar structural motifs and physicochemical properties. The significant reduction in ROS levels in both the liver and jejunum across all treatment groups at 21 days indicates a dual mechanism of oxidative damage mitigation, involving both direct radical scavenging and inhibition of ROS generation, as well as indirect enhancement of antioxidant enzyme-substrate binding efficiency through physical adsorption. By 42 days, while liver ROS showed no significant differences, D-pal maintained significantly reduced ileal ROS levels, suggesting systemic antioxidant adaptation to chronic supplementation. Furthermore, transcriptomic analyses revealed that Pal sustains ROS homeostasis during oxidative stress by modulating gene expression networks, particularly the Nrf2-ARE pathway. Zinc ions are essential for metallothionein (MT) induction. The MT directly scavenges ROS via its 20-21 sulfhydryl groups (-SH) per molecule [57,58]. Simultaneously, Zn^2+^ serves as an indispensable cofactor for GSH-Px, directly facilitating enzymatic activation. Moreover, Pal’s acicular crystal structure increases specific surface area, enabling adsorption of enteric endotoxins (e.g., *E. coli* lipopolysaccharide), thereby reducing oxidative damage to hepatocytes and intestinal mucosa [25]. Zinc further confers anti-inflammatory and antioxidant activities through multiple pathways [59,60,61].

In the duodenum of all treatment groups, the activities of SOD and CAT were significantly elevated. This observation aligns with prior findings from our laboratory, demonstrating that Pal adsorption of enteric pathogens and toxins reduces localized oxidative stress, with preferential effects observed in the duodenum [25]. In jejunal and ileal segments, D-pal significantly enhanced CAT activity in the jejunum, while N-pal reduced MDA levels in the ileum. These findings suggest distinct modulation mechanisms by different mineral morphologies—either through targeted activation of specific antioxidant enzymes (e.g., CAT upregulation in the jejunum) or inhibition of lipid peroxidation cascades (e.g., MDA reduction). These results demonstrate that Pal alleviates oxidative stress by enhancing the endogenous antioxidant defense system. At 42 days, only the D-pal group maintained significantly elevated SOD activity in the ileum, indicating sustained antioxidant effects. This long-term efficacy may be attributed to the structural stability of the mineral phase. The dispersed D-pal enhances adsorption capacity and apparent antioxidant activity through dissociation of crystal bundles, which exposes more active sites. This suggests that physical modification (e.g., bundle-dissociation) is an effective strategy to improve the functional performance of mineral additives. Furthermore, the zinc-loading design achieves a synergistic effect by concurrently boosting antioxidant capacity and supplementing trace elements, thereby reducing the required dosage of inorganic zinc in feed. This approach is consistent with the evolving practices in sustainable poultry production.

Transcriptomics has been widely applied in poultry antioxidant research to explore the molecular mechanisms of oxidative stress and identify key regulatory genes and pathways. N-pal exerts fundamental antioxidant effects primarily via glutathione metabolism and immune network pathways [62], aligning with the established “toxicant sequestration” mechanism of mineral adsorbents [63,64]. Furthermore, GSEA demonstrated significant downregulation of the “response to oxidative stress” pathway, suggesting that N-pal likely functions by reducing pro-oxidative stimuli rather than directly activating antioxidant enzymes. The activation of the PPAR pathway in D-pal group represents a critical breakthrough. PPARα, as a key regulator of lipid metabolism, orchestrates the expression of antioxidant enzymes—consistent with the significant enrichment of oxidoreductase activity observed in the D-pal group [65]. The substantially increased specific surface area likely facilitates enhanced binding of fatty acid ligands to PPAR receptors, extending beyond mere adsorption to direct metabolic regulation. The mechanism of zinc-loaded attapulgite (Zn-pal) demonstrates the greatest complexity: Enrichment in MAPK and Wnt pathways implies zinc ions coordinate antioxidant responses through dual routes. Primarily, Zn^2+^ activates the Nrf2 pathway—the canonical antioxidant cascade—while concurrently, Wnt/β-catenin signaling maintains intestinal stem cell homeostasis, thereby enhancing oxidative stress resilience at the tissue architecture level [66]. The downregulation of “cellular response to hydrogen peroxide” revealed by GSEA directly accounts for its superior MDA reduction efficacy. Previous research indicates that zinc from mineral carriers exhibits higher bioavailability than inorganic zinc sources (e.g., zinc sulfate), enabling more effective activation of antioxidant pathways [25]. The Nrf2 pathway likely responds to subthreshold oxidative stress, as this system is known to be activated by trace ROS levels. This mechanism aligns precisely with our finding that Zn-pal sustains ROS reduction exclusively in the jejunum [67]. Analysis of gene expression in the liver and jejunal mucosa of broilers revealed that Pal modulates the expression of antioxidant genes through the Nrf2/HO-1 signaling pathway. In the N-pal group, toxin adsorption reduced oxidative substrates, indirectly downregulating genes in the “response to oxidative stress” pathway. However, it failed to significantly activate key Nrf2 downstream targets (e.g., *GPX1*). Conversely, both D-pal and Zn-pal groups activated the Nrf2 signaling pathway, upregulating HO-1, *SOD1*, and related genes to promote antioxidant enzyme synthesis. Specifically, Zn-pal established a self-reinforcing antioxidant feedback loop wherein zinc ions activated the MAPK pathway to enhance Nrf2 nuclear translocation. Notably, no significant changes in antioxidant gene expression were observed at 42 days, likely attributable to the short-term dynamics of transcriptional regulation and compensatory adaptations within the endogenous antioxidant system.

## 6. Conclusions

This study demonstrates that N-pal, D-pal, and Zn-pal exhibit scavenging capacity for H_2_O_2_ and ·OH. Dietary supplementation with N-pal, D-pal, and Zn-pal enhances the activities of antioxidant enzymes (GSH-Px, CAT, and SOD) and activates the Nrf2 signaling pathway to upregulate antioxidant-related genes in broilers. These effects are particularly pronounced in broilers at 21 days of age, with D-pal and Zn-pal demonstrating superior growth performance. Future research should prioritize exploring synergistic mechanisms of combined modification approaches and evaluating long-term impacts on the gut microbiome.

## Figures and Tables

**Table 1:** Compositions and nutrient levels of the experimental basal diet (as-fed basis, %).

Items	Starter (1–21 d)	Grower (22–42 d)
Ingredients, %		
Corn	57.00	61.50
Soybean meal	31.50	27.50
Corn gluten meal	3.55	3.00
Soybean oil	3.00	3.60
Limestone	1.20	1.23
Dicalcium phosphate	2.00	1.50
L-Lysine	0.30	0.25
DL-Methionine	0.15	0.12
Sodium chloride	0.30	0.30
Premix ^1^	1.00	1.00
Total	100.00	100.00
Calculated nutrient levels		
Apparent metabolizable energy, MJ/kg	12.36	12.68
Crude protein, %	21.91	20.15
Calcium, %	0.97	0.86
Total phosphorus, %	0.68	0.59
Available phosphorus, %	0.40	0.32
Lysine, %	1.25	1.11
Methionine, %	0.51	0.45
Methionine + cystine, %	0.86	0.80
Analyzed nutrient levels ^2^		
Crude protein, %	21.89	20.13
Calcium, %	0.95	0.85
Total phosphorus, %	0.68	0.58
Lysine, %	1.24	1.11
Methionine, %	0.50	0.45

^1^ Premix provided per kilogram of diet: vitamin A, 12,000 IU; vitamin D_3_, 3000 IU; vitamin E, 30 IU; vitamin K, 1.3 mg; thiamin, 2.2 mg; riboflavin, 8 mg; nicotinamide, 40 mg; choline chloride, 600 mg; calcium pantothenate, 10 mg; biotin, 0.04 mg; folic acid, 1 mg; vitamin B_12_, 0.013 mg; Fe (ferrous sulfate), 80 mg; Cu (copper sulphate), 8.0 mg; Mn (manganese sulphate), 110 mg; Zn (zinc oxide), 60 mg; I (calcium iodate), 1.1 mg; Se (sodium selenite), 0.3 mg. ^2^ Results are the average values of triplicate measurements.

**Table 2:** Sequences for real-time quantitative PCR primers.

Genes ^1^	Primer Sequence (5′–3′)	Gene Bank ID
β-actin	F TTGGTTTGTCAAGCAAGCGG R CCCCCACATACTGGCACTTT	NM_205518.1
SOD1	F CCGGCTTGTCTGATGGAGAT R TGCATCTTTTGGTCCACCGT	NM_205064.2
GPX1	F AATTCGGGCACCAGGAGAAC R TGTACTGCGGGTTGGTCATC	NM_001277853.3
HO-1	F GGTCCCGAATGAATGCCCTTG R ACCGTTCTCCTGGCTCTTGG	NM_205344.2
Nrf2	F GATGTCACCCTGCCCTTAG R CTGCCACCATGTTATTCC	NM_205117.1
CAT	F GTCTGACAACCAAGGTGGCG R TGAAACGCTGCACATCTCCT	NM_001031215.2

^1^ GPX1, glutathione peroxidase 1; HO-1, heme oxygenase-1; Nrf2, nuclear factor-erythroid 2-related factor-2; SOD1, superoxide dismutase 1; CAT, catalase.

**Table 3:** Effects of Pal and its derivatives on growth performance of broilers from 1 to 42 days of age.

Items ^1^	Groups				SEM ^2^	*p*-Value
	CON	Pal	D-pal	Zn-pal		
1~21d						
ABW (g)	812.56 ^c^	833.06 ^bc^	855.35 ^ab^	881.78 ^a^	7.22	0.002
ADG (g/d)	36.73 ^c^	37.71 ^bc^	38.77 ^ab^	40.01 ^a^	0.34	0.002
ADFI (g/d)	55.87	55.95	57.45	59.32	0.56	0.090
F/G (g:g)	1.48 ^a^	1.42 ^b^	1.41 ^b^	1.40 ^b^	0.01	0.046
21~42d						
ABW (g)	2649.64	2702.81	2734.53	2738.28	41.70	0.879
ADG (g/d)	87.48	89.04	89.48	88.41	1.97	0.987
ADFI (g/d)	181.42	184.55	181.50	177.58	2.31	0.784
F/G (g:g)	2.08	2.09	2.06	2.04	0.04	0.962
1~42d						
ADG (g/d)	62.11	63.38	64.13	64.21	0.99	0.880
ADFI (g/d)	117.87	119.06	118.11	116.87	1.14	0.934
F/G (g:g)	1.90	1.89	1.85	1.83	0.02	0.734

^a,b,c^ Means within the same row without a common superscript differ significantly (*p* < 0.05). ^1^ ABW, average body weight; ADG, average daily weight gain; ADFI, average daily feed intake; F/G, feed-to-gain ratio; ^2^ SEM, standard error of the mean.

**Table 4:** Effect of adding Pal and its derivatives to the diet on the antioxidant enzyme activities in the serum and liver of broilers at 21 days.

Items ^1^	Groups				SEM	*p*-Value
	CON	N-pal	D-pal	Zn-pal		
Serum						
T-AOC (U/mL)	0.46	0.68	0.62	0.54	0.03	0.123
GSH-Px (U/mL)	659.39 ^c^	728.07 ^a^	693.22 ^ab^	681.23 ^ab^	8.77	0.036
SOD (U/mL)	401.45 ^b^	454.35 ^a^	463.25 ^a^	452.20 ^a^	8.76	0.044
CAT (U/mL)	2.21	2.45	2.28	2.61	0.08	0.213
MDA (nmol/mL)	3.33 ^a^	2.62 ^b^	2.35 ^b^	2.31 ^b^	0.12	0.004
Liver						
T-AOC (U/mg protein)	0.41	0.75	0.70	0.32	0.04	0.000
GSH-Px (U/mg protein)	20.64	24.09	24.90	23.24	1.06	0.540
SOD (U/mg protein)	33.44	34.23	34.56	34.37	0.34	0.685
CAT (U/mg protein)	3.78	3.85	3.90	3.73	0.11	0.954
MDA (nmol/mg protein)	1.11 ^a^	0.97 ^b^	0.87 ^bc^	0.77 ^c^	0.03	<0.001

^a,b,c^ Means within the same row without a common superscript differ significantly (*p* < 0.05). ^1^ T-AOC, total antioxidant capacity; GSH-Px, glutathione peroxidase; SOD, superoxide dismutase; CAT, catalase; MDA, malondialdehyde. SEM, Standard Error of the Mean.

**Table 5:** Effect of adding Pal and its derivatives to the diet on the antioxidant enzyme activities in the duodenum, jejunum, and ileum of broilers at 21 days.

Items ^1^	Groups				SEM	*p*-Value
	CON	N-pal	D-pal	Zn-pal		
Duodenum						
T-AOC (U/mg protein)	0.77	0.73	0.61	0.68	0.03	0.373
GSH-Px (U/mg protein)	18.44	17.46	23.79	22.77	1.55	0.400
SOD (U/mg protein)	24.18 ^c^	42.17 ^a^	44.67 ^a^	33.74 ^b^	1.59	<0.001
CAT (U/mg protein)	1.06 ^b^	1.85 ^a^	1.75 ^a^	1.53 ^ab^	0.11	0.029
MDA (nmol/mg protein)	0.75	0.64	0.45	0.51	0.06	0.229
Jejunum						
T-AOC (U/mg protein)	0.70	0.70	0.84	0.78	0.03	0.216
GSH-Px (U/mg protein)	11.52	14.45	11.98	15.86	1.97	0.859
SOD (U/mg protein)	42.61	39.49	39.04	44.49	1.16	0.301
CAT (U/mg protein)	1.10 ^b^	1.88 ^a^	1.71 ^a^	1.64 ^a^	0.10	0.015
MDA (nmol/mg protein)	1.13	1.09	1.13	1.05	0.05	0.925
Ileum						
T-AOC (U/mg protein)	0.31	0.19	0.22	0.22	0.03	0.398
GSH-Px (U/mg protein)	6.54	6.41	6.70	6.25	0.62	0.996
SOD (U/mg protein)	25.69 ^c^	36.88 ^b^	40.52 ^a^	25.58 ^c^	1.23	<0.001
CAT (U/mg protein)	1.27 ^b^	1.76 ^a^	1.56 ^ab^	1.36 ^b^	0.06	0.011
MDA (nmol/mg protein)	0.55 ^a^	0.37 ^b^	0.42 ^ab^	0.30 ^b^	0.03	0.019

^a,b,c^ Means within the same row without a common superscript differ significantly (*p* < 0.05). ^1^ T-AOC, total antioxidant capacity; GSH-Px, glutathione peroxidase; SOD, superoxide dismutase; CAT, catalase; MDA, malondialdehyde. SEM, Standard Error of the Mean.

**Table 6:** Effect of adding Pal and its derivatives to the diet on the antioxidant enzyme activities in the serum and liver of broilers at 42 days.

Items ^1^	Groups				SEM	*p*-Value
	CON	N-pal	D-pal	Zn-pal		
Serum						
T-AOC (U/mL)	0.61	0.66	0.50	0.77	0.04	0.054
GSH-Px (U/mL)	1035.77	1026.94	1034.96	995.27	9.32	0.391
SOD (U/mL)	386.06	409.29	452.17	450.43	18.89	0.551
CAT (U/mL)	2.25	2.63	2.38	2.7	0.09	0.294
MDA (nmol/mL)	4.55 ^a^	3.69 ^bc^	3.93 ^bc^	3.40 ^c^	0.11	<0.001
Liver						
T-AOC (U/mg protein)	0.45	0.50	0.45	0.42	0.02	0.658
GSH-Px (U/mg protein)	14.29	15.10	14.52	14.34	1.61	0.980
SOD (U/mg protein)	21.92	22.62	22.54	23.92	0.42	0.402
CAT (U/mg protein)	2.84	2.99	2.78	2.81	0.11	0.925
MDA (nmol/mg protein)	1.65	1.49	1.49	1.33	0.05	0.120

^a,b,c^ Means within the same row without a common superscript differ significantly (*p* < 0.05). ^1^ T-AOC, total antioxidant capacity; GSH-Px, glutathione peroxidase; SOD, superoxide dismutase; CAT, catalase; MDA, malondialdehyde. SEM, Standard Error of the Mean.

**Table 7:** Effect of adding Pal and its derivatives to the diet on the antioxidant enzyme activities in the duodenum, jejunum, and ileum of broilers at 42 days.

Items ^1^	Groups				SEM	*p*-Value
	CON	N-pal	D-pal	Zn-pal		
Duodenum						
T-AOC (U/mg protein)	0.58	0.54	0.50	0.50	0.03	0.651
GSH-Px (U/mg protein)	17.24	14.63	15.98	17.81	0.97	0.318
SOD (U/mg protein)	15.76	21.62	18.61	21.64	1.35	0.369
CAT (U/mg protein)	1.17	1.30	1.11	1.13	0.16	0.981
MDA (nmol/mg protein)	1.01	0.90	0.91	0.82	0.05	0.686
Jejunum						
T-AOC (U/mg protein)	0.54	0.52	0.65	0.59	0.03	0.272
GSH-Px (U/mg protein)	11.43	12.63	10.45	12.30	1.52	0.982
SOD (U/mg protein)	33.29	28.70	29.30	31.41	1.28	0.593
CAT (U/mg protein)	0.94	1.20	1.10	1.13	0.11	0.871
MDA (nmol/mg protein)	1.39	1.25	1.19	1.22	0.05	0.499
Ileum						
T-AOC (U/mg protein)	0.25	0.26	0.24	0.25	0.03	0.990
GSH-Px (U/mg protein)	5.90	5.62	5.72	6.20	0.55	0.605
SOD (U/mg protein)	23.15 ^b^	24.60 ^b^	26.94 ^a^	23.41 ^b^	0.32	<0.001
CAT (U/mg protein)	1.13	1.26	1.21	1.19	0.09	0.978
MDA (nmol/mg protein)	0.72 ^a^	0.60 ^ab^	0.56 ^b^	0.55 ^b^	0.02	0.034

^a,b^ Means within the same row without a common superscript differ significantly (*p* < 0.05). ^1^ T-AOC, total antioxidant capacity; GSH-Px, glutathione peroxidase; SOD, superoxide dismutase; CAT, catalase; MDA, malondialdehyde. SEM, Standard Error of the Mean.

## Data Availability

Transcriptional data presented in this work have been deposited in the National Center for Biotechnology Information (NCBI) (https://www.ncbi.nlm.nih.gov, accessed on 1 August 2025) and accessible through NCBI number SUB15437676. All other data will be made available on reasonable request.

## Abbreviations

The following abbreviations are used in this manuscript:

Pal	Palygorskite
N-pal	Natural palygorskite
·OH	Hydroxyl radicals
BP	Biological process
CAT	Catalase
CC	Cellular component
CON	Control
cDNA	Complementary DNA
DEGs	Differentially expressed genes
DHE	Dihydroethidium
D-pal	Bundle-dissociation palygorskite
FPKM	Fragments Per Kilobase per Million mapped fragments
GO	Gene Ontology
GPX1	Glutathione peroxidase 1
GSEA	Gene Set Enrichment analysis
GSH-Px	Glutathione peroxidase
H_2_O_2_	Hydrogen peroxide
HO-1	Heme oxygenase-1
KEGG	Kyoto Encyclopedia of Genes and Genomes
MF	Molecular function
Nrf2	Nuclear factor-erythroid 2-related factor-2
qRT-PCR	Quantitative Real-Time PCR
RNA-seq	Transcriptome sequencing
RNS	Reactive nitrogen species
ROS	Reactive oxygen species
SEM	Standard error of the mean
SOD	Superoxide dismutase
SOD1	Superoxide dismutase 1
T-AOC	Antioxidant capacity
Zn-pal	Zinc-bearing palygorskite
